# Sense and Antisense Transcripts of Convergent Gene Pairs in *Arabidopsis thaliana* Can Share a Common Polyadenylation Region

**DOI:** 10.1371/journal.pone.0016769

**Published:** 2011-02-02

**Authors:** Elena Zubko, Andrea Kunova, Peter Meyer

**Affiliations:** University of Leeds, Centre for Plant Sciences, Leeds, United Kingdom; Oregon State University, United States of America

## Abstract

The *Arabidopsis* genome contains a large number of gene pairs that encode sense and antisense transcripts with overlapping 3′ regions, indicative for a potential role of natural antisense transcription in regulating sense gene expression or transcript processing. When we mapped poly(A) transcripts of three plant gene pairs with long overlapping antisense transcripts, we identified an unusual transcript composition for two of the three gene pairs. Both genes pairs encoded a class of long sense transcripts and a class of short sense transcripts that terminate within the same polyadenylation region as the antisense transcripts encoded by the opposite strand. We find that the presence of the short sense transcript was not dependent on the expression of an antisense transcript. This argues against the assumption that the common termination region for sense and antisense poly(A) transcripts is the result of antisense-specific regulation. We speculate that for some genes evolution may have especially favoured alternative polyadenylation events that shorten transcript length for gene pairs with overlapping sense/antisense transcription, if this reduces the likelihood for dsRNA formation and transcript degradation.

## Introduction

Animal and plant genomes contain a surprisingly large number of partly overlapping convergent gene pairs representing approximately 7.5% of all protein-encoding genes in Arabidopsis [1]; [2]; [3]. Bidirectional transcription of both genes can lead to the formation of dsRNA substrates for RNA interference mechanisms that involve DICER-mediated cleavage and small RNA production [4]; [5]. In plants, mainly RNA interference-based antisense effects have been described [4]; [6]; [7]; [8] while in animal and yeast systems we find a variety of.

Bidirectional transcription of two yeast genes causes transcriptional interference between the two RNAII polymerase complexes affecting transcript elongation and termination [9]. In mammals, antisense transcription through promoter regions can interfere with transcriptional initiation and can alter DNA methylation patterns. In many imprinted genes, antisense transcription from CpG islands within an imprinted gene leads to expression competition causing promoter methylation and silencing [10]. A similar effect was generated when chromosomal rearrangements created antisense transcription downstream of the (*HBA2*) α-globin gene, which induced methylation and silencing of the HBA2 promoter [11]. RNA-based promoter inactivation is not restricted to antisense transcripts as an example for sense-specific transcriptional interference has recently been reported in plants. A T-DNA insert that initiated the transcription of a large polycistronic transcript caused inactivation of transcriptional initiation at a gene located downstream [12].

Other regulatory effects based on antisense transcription include selective transcript editing by dsRNA-dependent adenosine deaminases (ADARs) and retention of hyperedited RNAs in the nucleus [13], antisense-mediated splice form selection [14]; [15] and antisense-based modulation of mRNA translation [16]. In addition to model genes, for which a direct effect of antisense transcription on a sense transcript has been demonstrated, some reports highlight the presence of partly overlapping sense and antisense transcripts at genomic loci as an indicator for antisense-mediated regulation [17].

To examine potential sense/antisense effects on polyadenylation sites in *Arabidopsis,* we examined three gene pairs that encode convergent transcripts with long overlapping 3′ region. Surprisingly, we detected in two gene pairs the same arrangement of two classes of polyadenylated sense transcripts, one of which shared a polyadenylation region with the antisense transcripts. Contrary to the expectation that antisense transcription or processing regulates alternative polyadenylation of the sense gene, we find that the two alternative sense transcript classes are independent of antisense transcription. The common presence of shortened transcripts in two sense/antisense gene pairs may be the result of evolutionary selection, if this reduces potentially negative effects from dsRNA formation.

## Results

### Selection of gene pairs with overlapping transcripts

**Figure 1:**
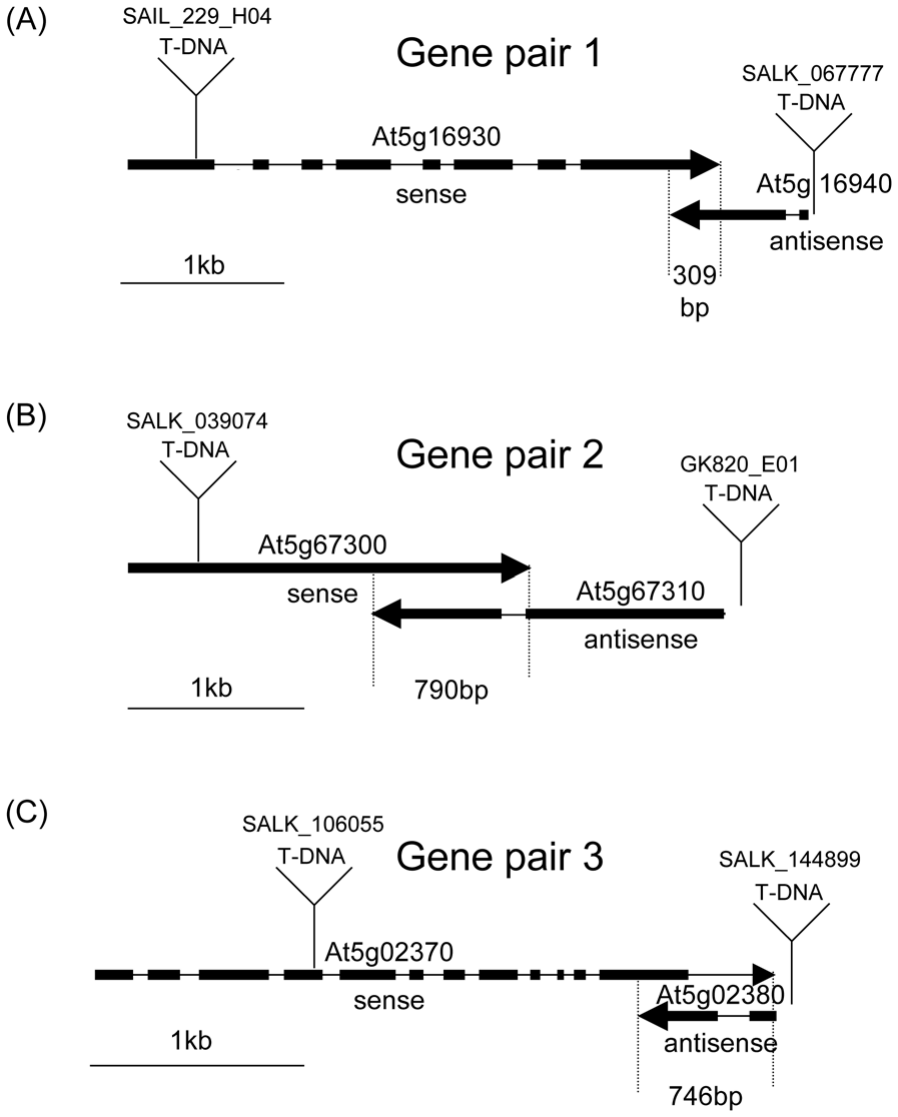
Maps of three gene pairs encoding sense and antisense transcripts with convergently overlapping 3′ ends (Convergently Overlapping Gene Pairs). Exons are labelled as tick lines, introns are drawn as thin lines. T-DNA insert positions are shown for each of the six mutant lines analysed. All distances are drawn to scale.

The Arabidopsis genome contains 956 pairs of coding genes that overlap at their 3′ ends [1], and that have been termed Convergently Overlapping Gene Pairs (COPs). Among these, we selected three gene pairs that, according to the transcript annotation on TAIR [18] have long overlapping regions. At5g16930 and At5g16940 transcripts share 309 bp of their 3′ regions (gene pair 1), At5g67300 and At5g67310 transcripts have a 790bp overlapping region (gene pair 2), and At5g02370 and At5g02380 transcripts share a 746 bp region (gene pair 3). The MPSS database (http://mpss.udel.edu/at/mpss_index.php) [19] identifies small RNAs for the three gene pairs. In contrast to genes with coding information that overlap with a non-coding antisense transcript, each COP member fulfils two functions. It encodes a sense gene and it provides an antisense transcript to the overlapping partner gene. To avoid confusions, we arbitrarily labelled the gene with the lowest Gene ID the sense gene and its partner gene the antisense gene. The overlapping regions were annotated to contain no introns in pair 1, one antisense transcript intron in pair 2, and introns in both transcripts of pair 3 (Figure 1). Pair 1 consists of the AT5G16930 gene encoding an AAA-type ATPase family protein and the AT5G16940 gene encoding a carbon-sulfur lyase. Pair 2 contains the AT5G67300 gene encoding a myb family transcription factor and the AT5G67310 gene encoding a cytochrome P450 family protein. Pair 3 consists of the AT5G02370 gene encoding a kinesin motor protein-related and the AT5G02380 gene encoding metallothionein protein 2B.

**Figure 2:**
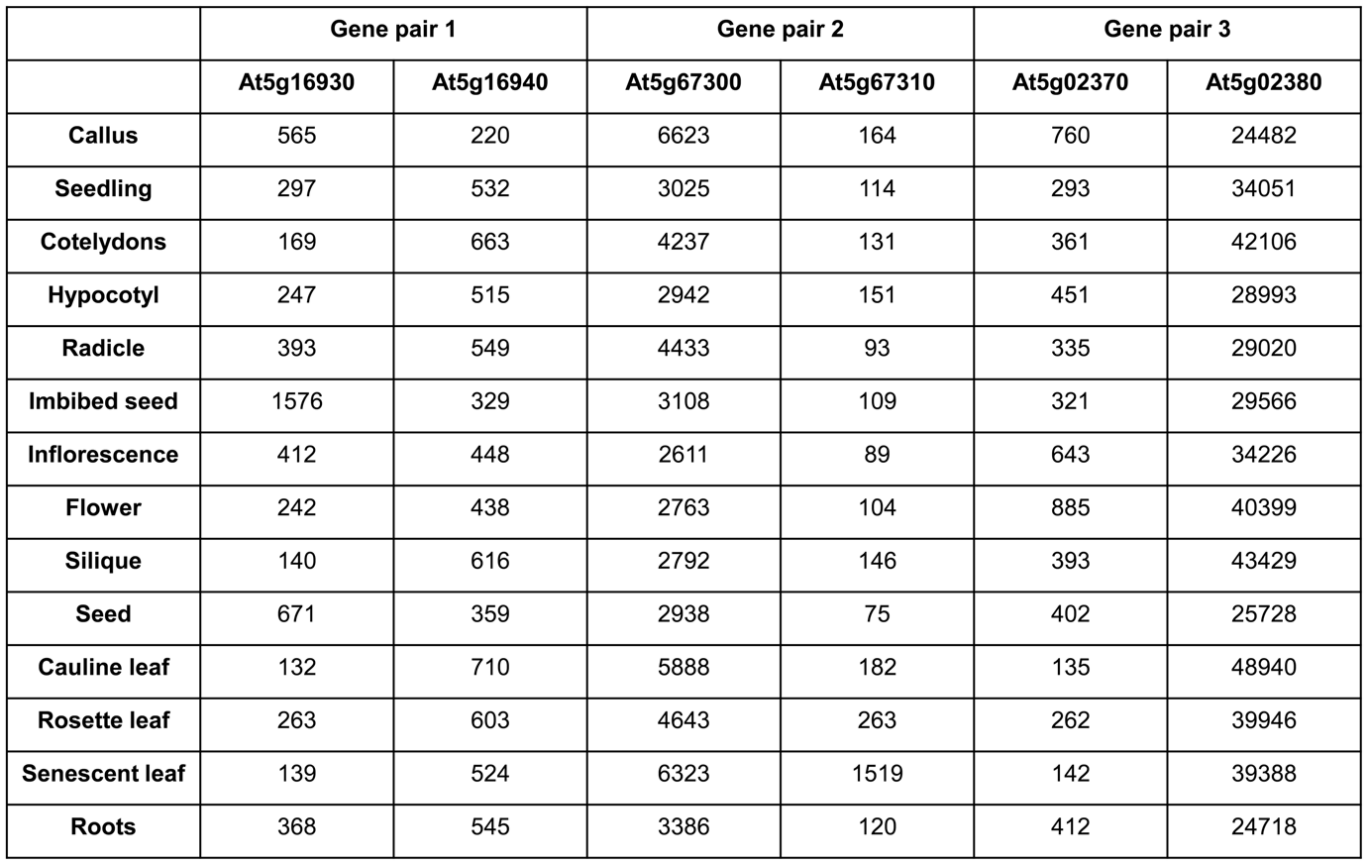
Sense and antisense transcript levels of the three gene pairs. Tissue-specific expression levels were extracted from Genevestigator as arbitrary signal intensity values. A value of 1000 represents the trimmed mean of all scaled signal values, the background range is in the range 0-100 [20].

Microarray data [20] show comparable expression levels for At5g16930 and At5g16940, while for the other two gene pairs expression levels of one gene (At5g67300 and At5g02380) are much higher than those of the corresponding partner. Gene pairs 2 and 3 show no antagonistic correlation in expression level that could suggest potential tissue specific antagonistic effects between sense and antisense. In contrast, the expression of the two genes of pair 1 anti-correlates in most tissues (Figure 2).

### Analysis of T-DNA lines with altered transcript levels

**Figure 3:**
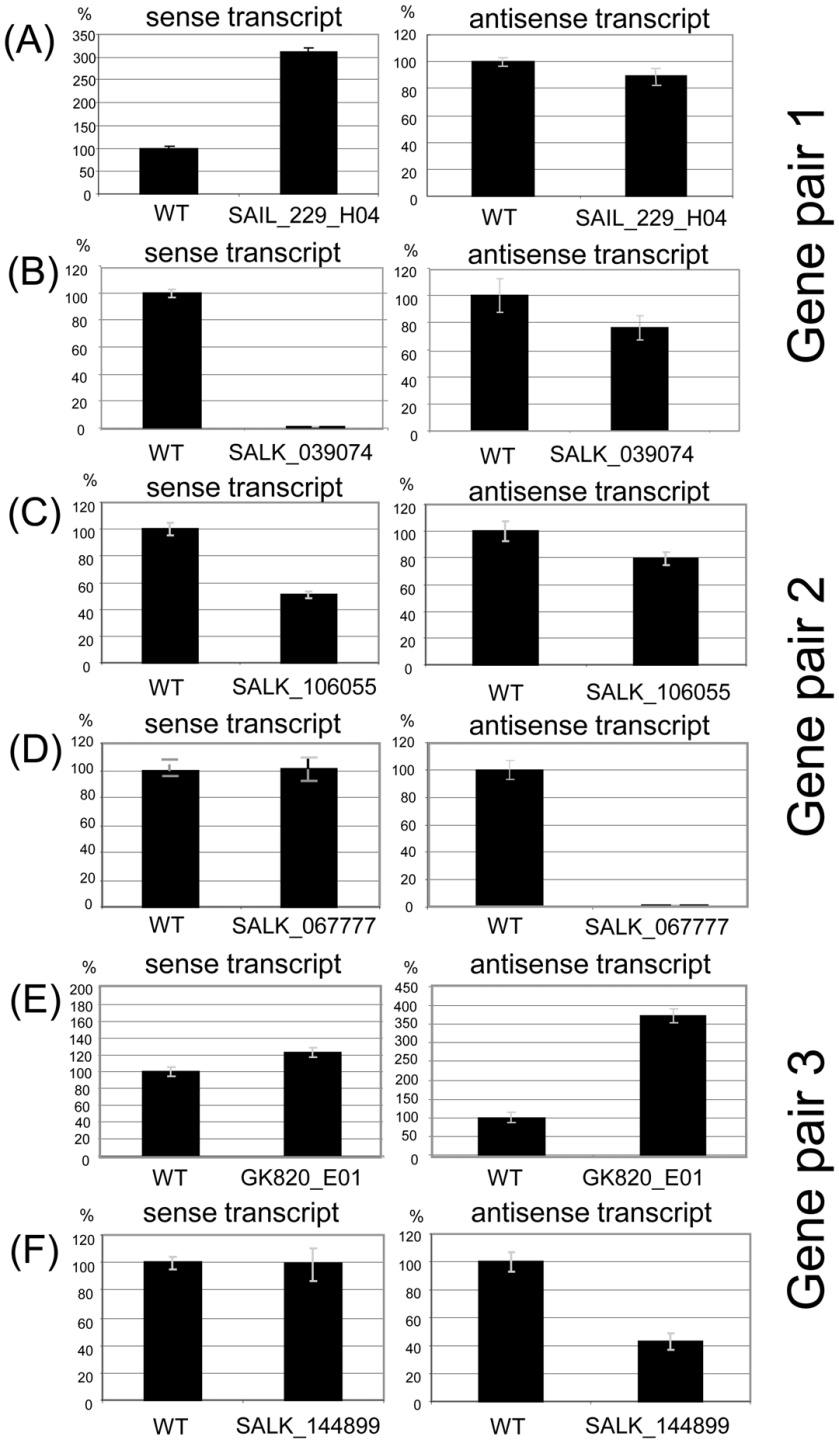
Q-PCR analysis of sense transcript levels in wildtype and mutant lines for the three loci shown in **figure 1**. (A–C) T-DNA insertions in sense genes can increase (A) or reduce (B, C) sense transcript levels. Antisense transcripts show no significant antagonistic response to altered transcript levels in sense gene insertion lines except for a moderate decrease of the antisense transcript in line SAIL_229_H04 with increased sense transcript levels. (D–F) T-DNA insertions in antisense genes can increase (E) or reduce (D, F) antisense transcript levels. Sense transcripts levels show no antagonistic response to altered transcript levels in antisense gene insertion lines. Error bars show standard deviation of mean values.

We assumed that the efficiency of any RNA interference effects between sense and antisense transcripts should be reduced if transcription of one of the genes is down-regulated. Equally, it should be enhanced if transcription of one of the genes is up-regulated. We therefore examined if T-DNA insertion lines for the six genes showed antagonistic changes in sense and antisense transcript levels. Depending on the activity of T-DNA promoter and enhancer elements, insertion lines can reduce or increase transcript levels downstream of the T-DNA integration site. The SAIL line contain a pCSA110 vector with a 1′2′ dual promoter near the left border, the GK line contain a pAC161 vector with a 35S promoter at its right border, and the SALK lines contain a pROK2 vector with a 35S promoter near the left border. These and other T-DNA-specific promoter fragments have the potential to induce read-through transcription [21] Steady-state transcript levels were reduced in four T-DNA lines and enhanced in two T-DNA lines. With the exception of a moderate effect on the antisense gene of pair 1 (Figure 3A), none of these lines showed antagonistic changes in transcript levels of the partner genes, which does not argue for RNA interference or other quantitative effects regulating transcript levels (Figure 3).

### A class of short sense transcripts that share a common polyadenylation region with antisense transcripts

**Figure 4:**
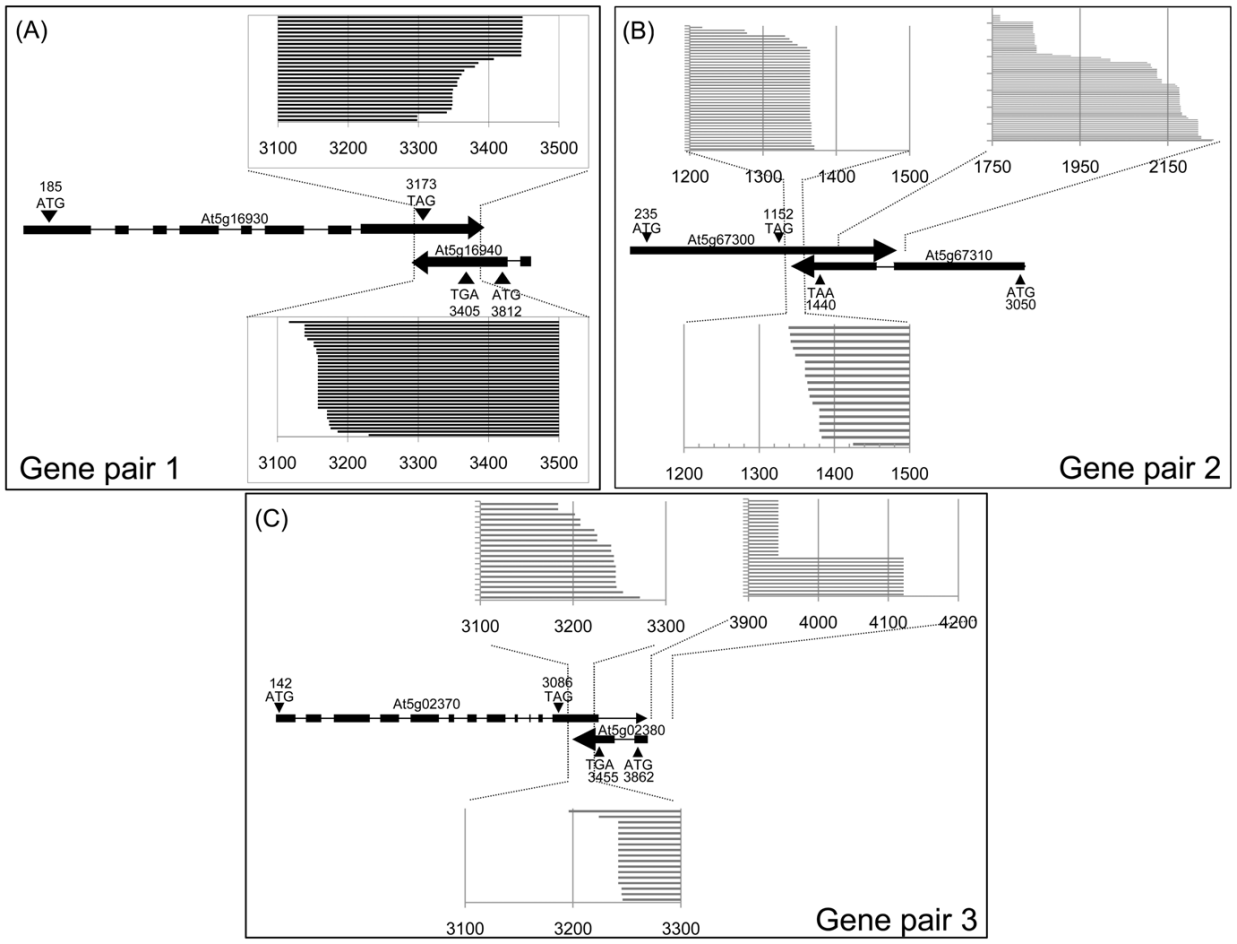
Polyadenylation of sense and antisense transcripts of COPs with two classes of sense transcripts. The figure compiles information about polyadenylation sites of sense and antisense transcripts of gene pairs At5g16930/40 (A), At5g67300/10 (B) and At5g02370/80 (C). Numbers refer to nucleotide positions relative to the transcriptional start site of the sense gene. Individual bars represent individual cloned poly(A) transcripts isolated from leaves, flowers, seedlings and seeds. The 3′ regions of sense transcripts are drawn as black bars from left to right, the 3′ regions of antisense transcripts are drawn as black bars from right to left. For the two gene pairs At5g67300/10 (B) and At5g02370/80 (C), we find a group of long transcripts and a group of short transcripts. Polyadenylation of the shorter transcripts occurs in the same region where antisense transcripts terminate.

**Figure 5:**
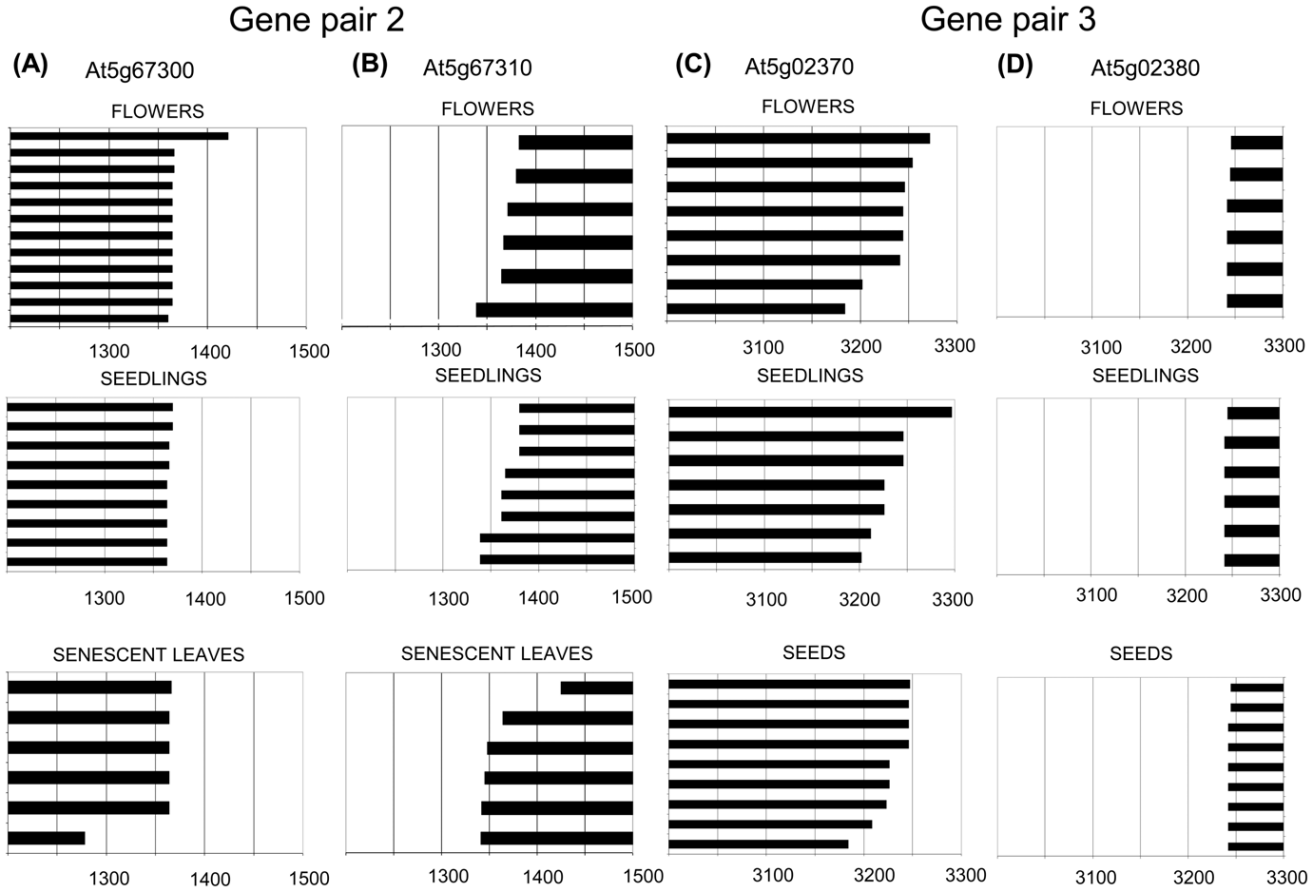
Polyadenylation sites of short transcripts (A,C) and antisense transcripts (B,D) of gene pairs At5g067300/10 and At5g02370/80 in different tissues. Short sense transcripts and antisense transcripts share a similar common polyadenylation region. Individual bars represent individual cloned poly(A) transcripts.

**Figure 6:**
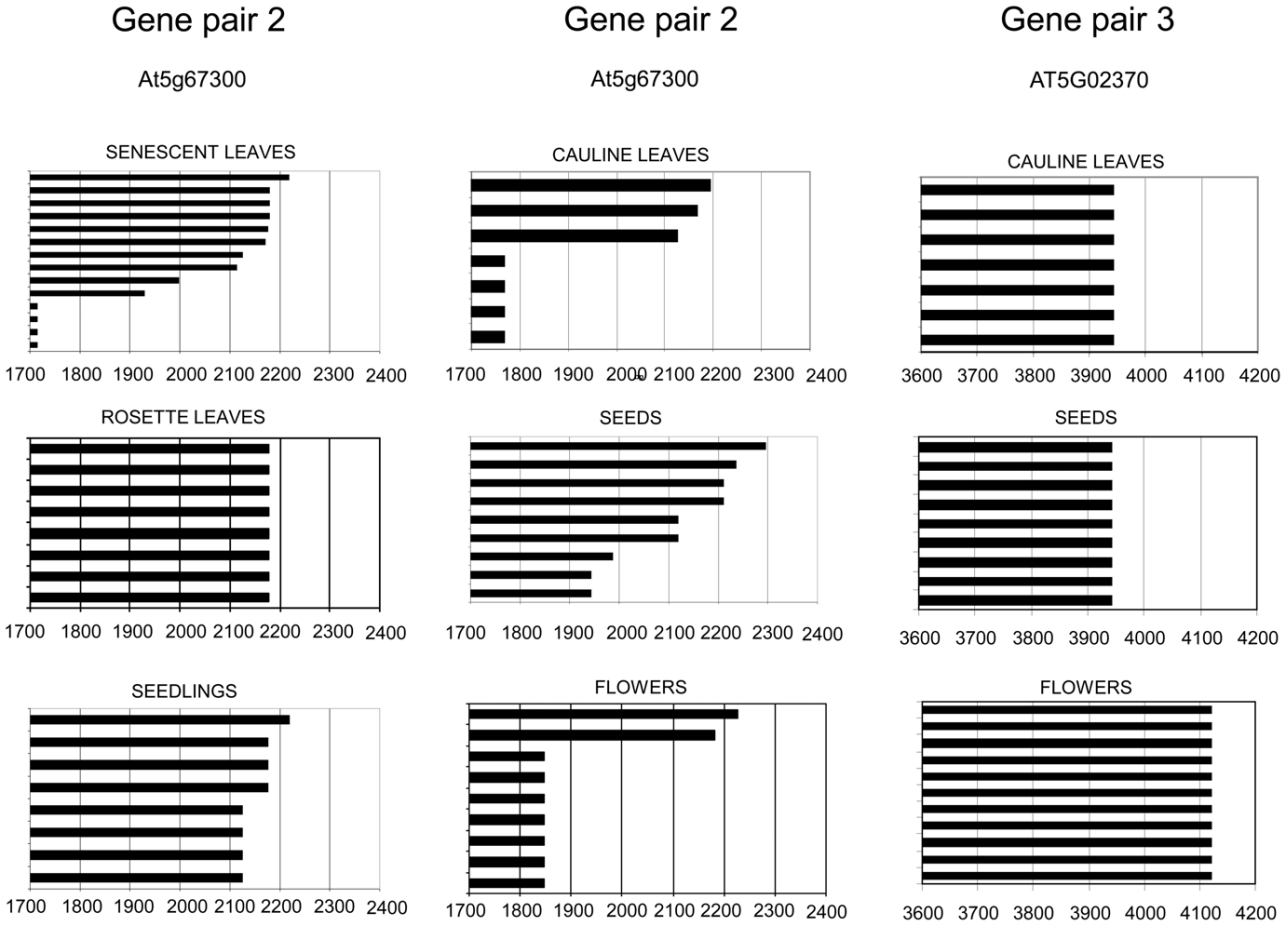
Tissue-specific variation of long polyadenylated At5g67300 and At5g02370 sense transcripts. Individual bars represent individual cloned poly(A) transcripts from different tissues. Numbers refer to nucleotide positions relative to the transcriptional start site.

**Figure 7:**
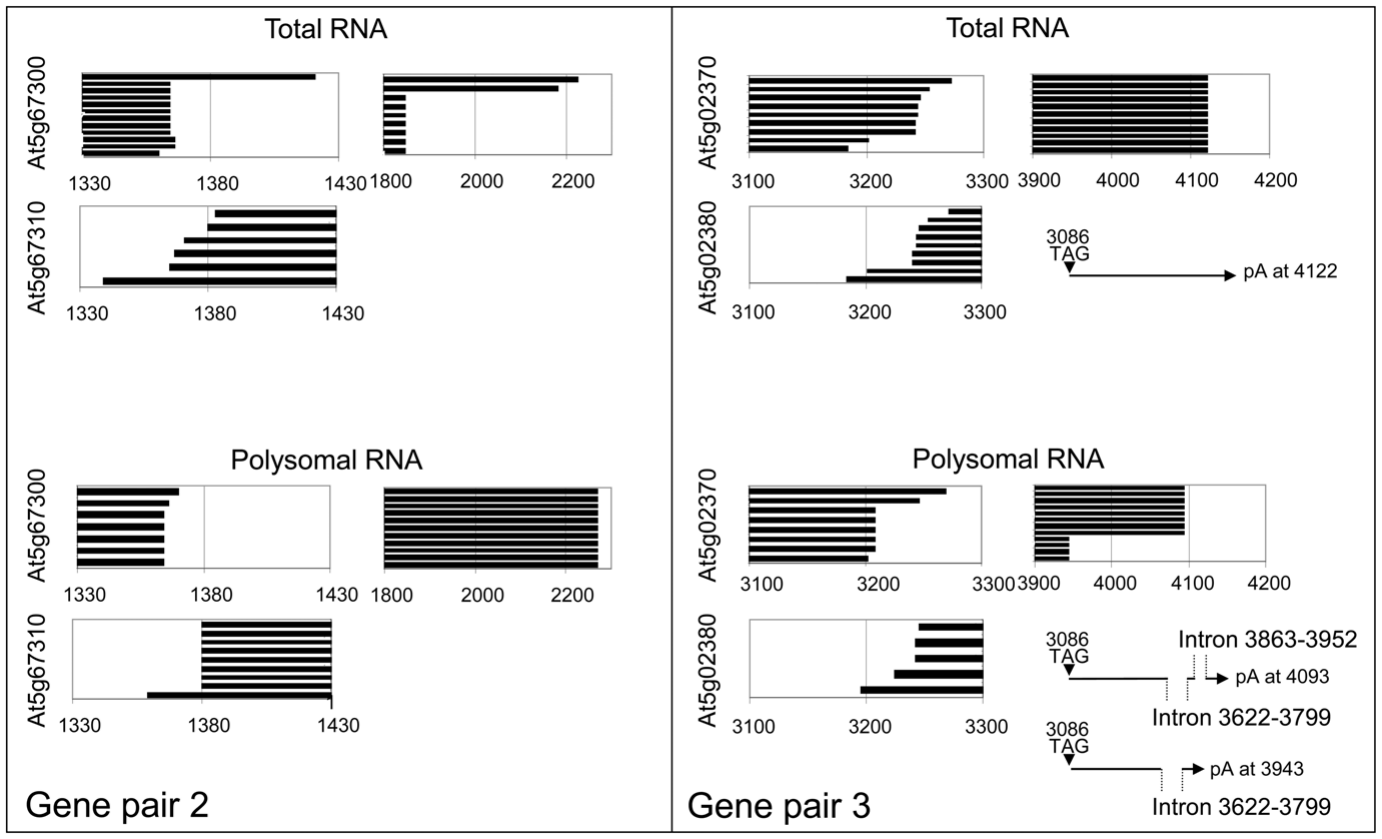
Comparison of polyadenylated At5g67300 and At5g02370 sense transcripts in total RNA and polysomal RNA fractions. Boxes contain the 3′ regions of sense and antisense transcripts for gene pair At5g67300/10 (left) and At5g02370/80 (right). 3′ regions of sense transcripts are drawn as black bars from left to right, 3′ regions of antisense transcripts are drawn as black bars from right to left. Individual bars represent individual cloned poly(A) transcripts from floral tissue. Below the boxes that show the 3′ region of long transcripts of At5g02370, the corresponding 3′UTR regions are depicted, to illustrate if the long transcript types were unspliced or spliced. Intron positions are inserted to scale. Polysomal fractions contain distinct classes of large sense transcripts that are not found in total RNA fractions. For At5g02370 transcripts, each polyadenylation class is linked to a distinct splicing pattern.

To assess if antisense transcription influenced polyadenylation of the sense transcript, or *vice versa*, we mapped poly(A) sites for the six genes (Figure 4). Transcripts of gene pairs 2 and 3 show a similar pattern with two size classes of sense transcripts, which we named short and long transcripts, respectively. For both gene pairs 2 and 3, alternative polyadenylation occurs within the 3′ UTR and does not affect the protein sequence. Exact quantitative comparison between short and long transcripts was difficult due to the use of different amplification primers, but for both gene pairs the short transcript was much more abundant than the long transcript as RT-PCR reaction for the long transcript required about 6 cycles more to amplify similar amounts as for the short transcript. While the polyadenylation region of the smaller transcripts is maintained in most tissues analysed (Figure 5), the size of the larger sense transcripts can vary by almost 200 bp in different tissues (Figure 6). A similar variability was detected for long sense transcripts in total and polysomal RNA preparations of the same tissue (Figure 7). We noticed, however, a difference in splicing between long At5g02370 sense transcripts isolated from total RNA and from polysomal RNA. None of the long At5g02370 sense transcripts of gene pair 3 that we cloned, contained the 3′UTR intron that is annotated in the TAIR database (Figure 4C). All transcripts isolated from a total RNA fraction terminated at the same poly(A) site and had a >1 kb long, intron-free 3′UTR region. In contrast, polysomal fractions only contained short sense transcripts, or long transcripts with intron deletions. Unspliced sense transcripts with a long 3′UTR were not detected in polysomal preparations (Figure 7). The spliced transcripts characteristic for polysomal RNA, were not detected in the total RNA fraction, which suggests that transcripts that are actually translated represent only a small subgroup of the mRNA population.

### The influence of antisense transcripts on sense transcript polyadenylation

**Figure 8:**
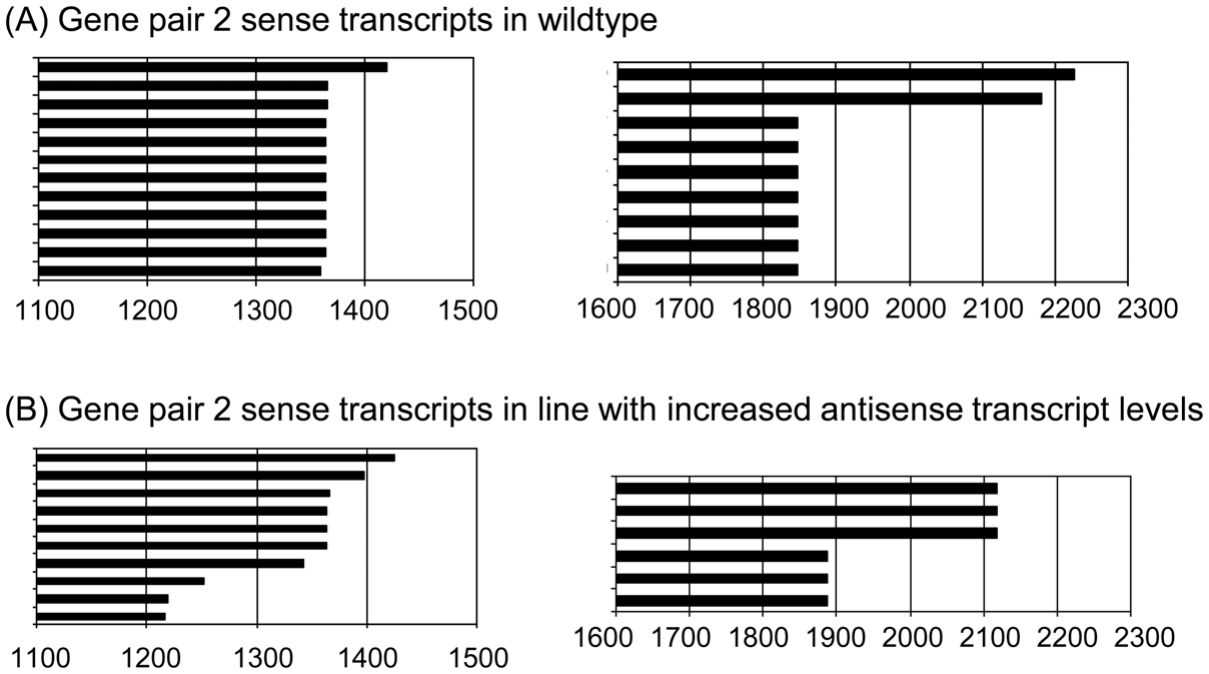
Analysis of At5g67300 short and long sense transcript polyadenylation in an insertion line for the antisense gene. Comparison of At5g67300 sense transcripts in wildtype (A) and in a T-DNA insertion line with increased antisense transcript levels (B), shows a shortening in some small sense transcripts, which may be a consequence of increased antisense transcript levels. Individual bars represent individual cloned poly(A) transcripts from floral tissue.

**Figure 9:**
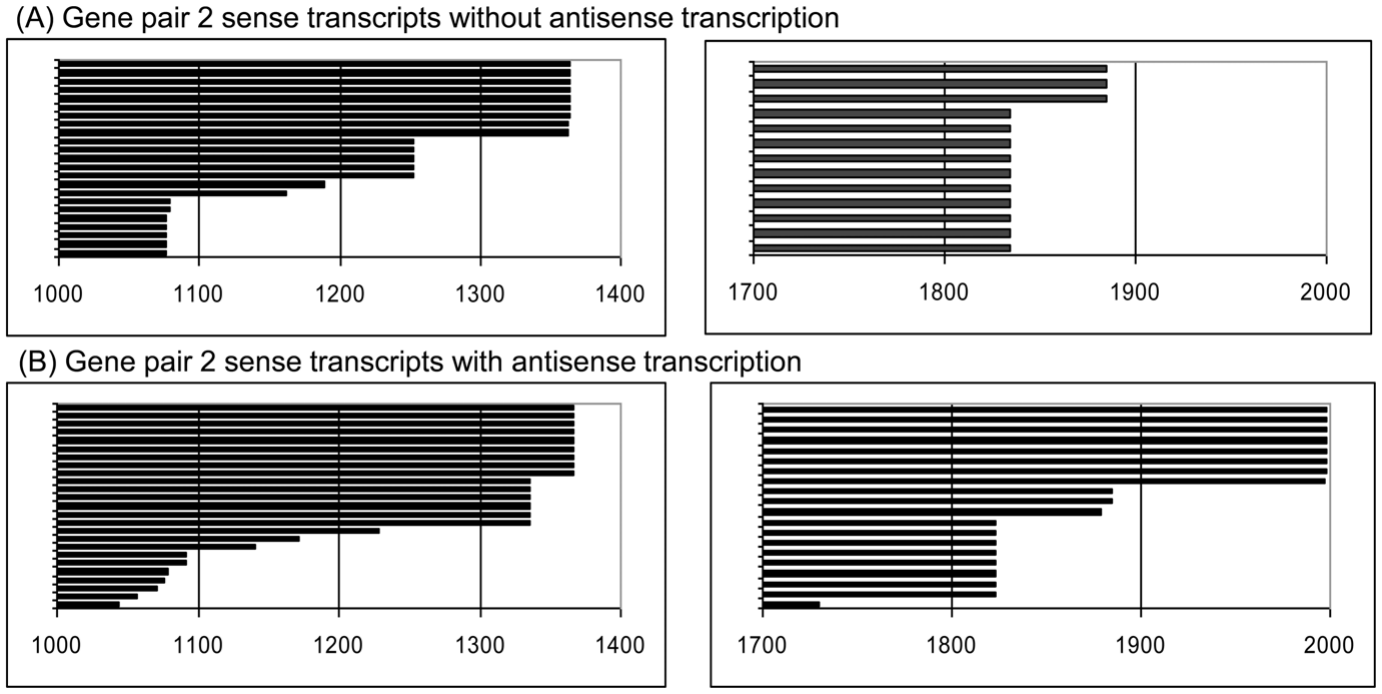
Short and long polyadenylated sense transcripts accumulate irrespective of the presence or absence of the antisense transcript. Comparison of At5g67300 sense transcripts in transient expression assays after transfer of recombinant constructs containing the At5g67300 sense gene in the presence (A) and absence of the antisense promoter (B). Boxes contain the 3′ regions of short and long At5g67300 sense transcripts. Individual bars represent individual cloned poly(A) transcripts from protoplasts.

To test if antisense transcription affected polyadenylation of small or large sense transcripts, we selected pair 2 comparing At5g67300 sense transcript polyadenylation in wildtype and in T-DNA insertion line GK820_E01, in which AT5g67310 antisense transcript levels are ∼4-fold enhanced (Figure 8). Wildtype and mutant lines contain a similar range of large sense transcripts. The short transcript did, however, show a higher level of variation in the mutant line, which may reflect moderate interference of antisense transcription and short transcript polyadenylation. To test if elimination of antisense transcription prevents early termination of the sense transcript, we designed two recombinant constructs with the At5g67300/At5g67310 gene pair linked to both the sense and antisense promoter, or only to the sense promoter, respectively. When transiently expressed in tobacco protoplasts, both constructs produce short and large sense transcripts (Figure 9). This does not support the assumption that antisense transcription is responsible for the early polyadenylation of the sense transcript. It rather suggests that polyadenylation of short sense transcripts and antisense transcripts occurs in the same genomic region, independent of the presence of an antisense transcript.

## Discussion

Natural antisense transcription has been proposed to comprise a second tier of gene expression in eukaryotes due to its influence on sense transcript synthesis, stability or processing [22]. In plants, RNA interference (RNAi) mechanisms control the degradation of dsRNA into small natural antisense transcript siRNAs (nat-siRNAs), which were first documented for the salt-induced degradation of the *P5CDH* gene, which encodes a stress-response regulator [4], and for the pathogen-induced repression of PPRL, a putative negative regulator of the RPS2 resistance pathway [6]. A more recent example is the *ARIADNE14* (*ARI14*) gene that encodes a putative ubiquitin E3 ligase and overlaps with the *KOKOPELLI (KPL)* gene. In sperm cells only, the overlapping transcripts generate a nat-siRNA pair, which regulates depletion of ARI14 transcripts, a prerequisite for double fertilisation [8]. The importance of RNAi effects is also documented by the presence of siRNAs matching the overlapping region of many sense and antisense genes with negatively correlated expression patterns [23]. RNAi effects should be responsive to quantitative changes in sense/antisense transcript ratios, but, except for a very small effect in gene pair 1, none of the three loci that were examined in this study showed significant antagonistic changes in sense or antisense transcript levels in T-DNA line where the concentration of the antagonistic partner transcript had been altered. This does not argue in favor of quantitative RNAi effects being involved in the regulation of these genes, but it does not exclude that these are limited to certain tissues or environmental conditions. We also cannot exclude that antisense transcription interferes with translation efficiency, or that degradation of dsRNA is restricted to specific cell types, developmental stages or cellular compartments. At least for one sense/antisense system, it has been documented that overlapping sense and antisense transcripts are degraded in the nucleus but unaffected in the cytoplasm [5]. While the three sense/antisense pairs may have the potential for RNA interference, we find no indication for antagonistic interactions of the transcript in our experimental system (Figure 3).

Non-quantitative effects of natural antisense transcripts have not been described for plant genes but there are examples of antisense transcripts in animals regulating RNA editing [13], RNA splicing [24] and translational regulation [25]. We detect some variation in polyadenylation sites for transcripts encoded by the three sense/antisense loci (Figure 5 and 6). The distribution of some polyadenylated transcripts varies in different tissues, which may indicate tissue-specific differences in the amount or specificity of transcript termination factors [26]. Long At5g02370 transcripts show a correlation between polyadenylation and splicing. Each of the three types of long polyadenylated transcripts has a characteristic splicing pattern with one, two or no introns being removed in the 3′UTR region (Figure 7). This confirms reports about a functional link between the splicing and 3′ end formation machineries in animals [27] and plants [28] by factors that are involved in both processes.

Total RNA preparations from floral tissue contain one type of long At5g02370 sense transcripts with an unusually long 3′ UTR. These transcripts are not detectable in polysomal fractions, which contain two types of long transcripts with shortened 3′ UTRs due to a combination of early polyadenylation and splicing. The 3′ UTRs of many mRNAs contain *cis*-acting elements that control RNA localisation and/or translation [29], and in many plant transcripts, 3′ UTRs longer than 300 nucleotides induce mRNA instability [30]. At5g02370 transcripts with long 3′ UTR may therefore be preferentially excluded from translation due to degradation, nuclear retention or other mechanisms that prevent their association with polysomes. Short transcripts, which are equally represented in total RNA and polysomal RNA fractions, would have a selective advantage over transcripts with long 3′ UTRs. This could have favoured the evolution of polyadenylation hot spots in upstream regions of 3′ UTRs.

Polyadenylation heterogeneity and alternative splicing [31] are frequently observed in animal [32] and plant transcripts [33]. The high level of alternative splicing may reflect variations in binding affinities of regulatory factors to 3′ UTRs influenced by variations in 3′ UTR structure or in polyadenylation complex proteins. RNA binding or processing factors have been identified that modify 3′ end formation of distinct mRNAs [34]; [35], and it has been suggested that differential polyadenylation complexes in different tissues are responsible for variable polyadenylation [36]. It was surprising that two of the three loci we examined showed a spatial correlation between polyadenylation sites of sense and antisense transcripts. While antisense transcription does not directly regulate sense transcript polyadenylation (Figure 8 and 9), the presence of an antisense transcript may have facilitated the evolution of alternative polyadenylation regions that generate shorter sense transcripts that are unable to form dsRNAs with antisense transcript and that would therefore have a selective advantage over larger sense transcript. A common phenomenon for both gene pairs is that sense and antisense genes have very different expression levels with one gene being expressed at relatively high levels and the partner gene being expressed at up to **∼**40 times lower levels (Figure 2). Genes with low expression levels might be particularly sensitive to RNAi effects if they are linked to an antisense gene with very high expression. Sense/antisense gene pairs with strong differences in expression may therefore be under specific selection pressure to prevent dsRNA formation.

As mentioned above, shortening of sense transcripts can also provide a selective advantage by improving translation of the transcript. Evolution may therefore have favoured premature polyadenylation of sense transcripts as a selective advantage over the synthesis of large sense transcripts with long 3′ UTRs, which are excluded from translation. This would apply to any gene with long 3′ UTRs, irrespective of the presence of an antisense transcript. If, however, pairing with overlapping antisense transcripts influenced translation efficiency of sense transcripts, sense/antisense gene pairs would especially benefit from alternative polyadenylation.

It is unclear which mechanism regulates polyadenylation of the short sense transcripts and the antisense transcripts within the same genomic region. If this effect was mediated by polyadenylation signals located within the overlapping polyadenylation region, these would be expected to be palindromic to ensure that they are equally represented on sense and antisense strands. In contrast to animal genes, however, there are no well defined consensus elements for polyadenylation of plant transcripts, and it has been suggested that secondary structures are involved in the recognition of polyadenylation regions [37] regulating variable polyadenylation [33]. The complementary 3′ regions of sense and antisense transcripts can be expected to form similar, although not identical, secondary structures. As an alternative to sequence-defined control elements, RNA folding might therefore be involved in facilitating polyadenylation of sense and antisense transcripts within a common region.

## Materials and Methods

### Genetic material

SALK insertion lines with T-DNA insertion in the coding sequence of At5g67300 (SALK_039074) and At5g67310 (SALK_106055), in the promoter sequence of At5g02380 (SALK_144899) and At5g16940 (SALK_067777) and the SAIL insertion line with T-DNA insertion in the coding sequence of At5g16930 (SAIL_229-H04) were obtained from the Nottingham Arabidopsis Stock Centre. The GK_820-E01 line with a T-DNA insertion in the promoter sequence of At5g67310 was obtained from the Gabi-Kat seeds collection (Bielefeld University, Germany).

### Plasmids construction

The At5g67300/At5g67310 sense/antisense construct was prepared in two steps. A 5788bp fragment containing both the sense and antisense gene and their promoter regions was amplified using Fhusion High-Fidelity DNA Polymerase (Finnzymes, New England Biolabs) with primers 2288 and 2236. PCR conditions were 98°C for 30 sec, and 30 cycles at 98°C for 10 sec, 58°C for 30 sec and 72°C for 3 min, followed by an elongation step at 72°C for 10 min. The PCR product was cloned into the pCR-Blunt vector (Invitrogen). The resulting construct was digested with *EcoR*I and the *EcoR*I fragment was inserted into the *EcoR*I site of vector pGreen0029.

For the preparation of the sense construct, a 5472 bp fragment was amplified using primers 2236 and 2130 at the same conditions described above, and the fragment was cloned into the pCR-Blunt vector (Invitrogen). The fragment, containing the At5g67300 sense promoter and At5g67300 sense gene was cut out with *EcoR*I and *Xmn*I, and was inserted into pGreen0029 that had been digested by *EcoR*I and *EcoR*V. The resulting construct lacks 198 bp of the 5′ region of the antisense gene and the **∼**300 bp antisense promoter region.

Plasmid DNA, isolated with the QIAGEN plasmid Midi Kit, was used in transient assays (see Table 1 for primer details).

**Table 1:** List of primer sequences in 5′ to 3′ orientation.

primer no	sequence
1160	GAGGTCTAACCCTGATGGAG
1164	CTCGTCTGAACAAAATCTGAG
1330	GCTGAGTCTAGATCACCAGG
1575	GGCCACGCGTCGACTAGTACT_(17)_
1576	GGCCACGCGTCGACTAGTAC
1818	CTCTCCTTGAGGCTCTTGACCAG
1819	CCAATACCACCAATCTTGTAGACATCC
1969	AGCAGCAGACAATTGAGATAAAAGGC
2015	TCGAGGATTGGTAAAGACTTGC
2018	TGGGCTTGAATCTTAATTGTG
2019	AACTTCATAAACCCTAAGTCTG
2116	AACGGAGGCGAGTTTATGGC
2118	GATGGCTGAGTACATCATTGAA
2129	CCTACAGGATTTGGCGGTAA
2130	TTCACGAATGGGTGAGATGA
2132	GGTGGATCATCGGAAGAAGA
2133	AAGCCCAACTGGATCTGATG
2197	TGCAGTTAGCTTCTCCAACC
2201	GAGAAAGACCAACGTCGCC
2214	CCAGGACATGCTCTCCTACC
2215	TACTTGTCCCACACGCTTCA
2216	TTGCACTTGCAGTCAGATCC
2217	CCACCGAGACTCTTGTCCTC
2218	CAAGGTCAAGGCACATCTCA
2219	ATTCTATGCGCGCTCTTCAT
2225	ATGTCGGAAGGAAAGGGTCT
2236	AGCGAGAACACGGAAGGATA
2288	AAGAGCTCCGAGCAAACAAC
2292	AGCCGATGCATTTCTGTGCG
2356	CAGCAATAATTAGGAATGGAATCAG
2357	AAAGTGAACATTGCCTCTCATTG

### RNA and cDNA preparation

Total RNA was isolated using a LiCl protocol [38] and treated with TURBO DNase (Ambion). cDNA was prepared from DNA-free RNA using Superscript II Reverse Transcriptase (Invitrogen) according to the manufacturer's recommendation.

### qRT-PCR

The qPCR analysis was performed uptsing a SensiMix™ SYBR&Fluorescein Kit from Quantace. Expression of the At5g16930/At5g16940 gene pair in seedlings was analysed using primers 2292 with 1330 for the sense gene and 2357 with 2356 for the antisense gene. At5g67300 sense transcripts levels were analysed in flowers with primers 2132 and 2133, and for At5g67310 antisense gene primers 2129 and 2130 were used. The At5g02370/At5g02380 gene pair was analysed in flowers using primers 2218 with 2219 and 2216 with 2217 respectively. All samples were calibrated according to the expression of the EF1α gene that was analysed using primers 1818 and 1819.

### Genotyping of insertion lines

Genomic DNA for genotyping was extracted from 3–4 weeks old leaf tissue according to [39]. After 1 hour incubation in the extraction buffer samples were cleaned up with 1V phenol:chloroform:IAA (12∶12∶1). Sequence data were obtained from TAIR [18]. The presence of the T-DNA insertions and homozygosity of insertion lines was assessed by two PCR reactions using the GoTaq master mix (Promega). A first PCR was performed with the forward and reverse gene specific primers, and in a second PCR an appropriate gene specific primer (forward or reverse) was used together with the following T-DNA insertion specific primer (Table 2).

**Table 2:** Gene-specific primers.

Gene	Insertion mutant tested	Forward primer	Reverse Primer	Size of the fragment
At5g02370	SALK_106055	TGATGCCATTGACCATGTCC	ATTCTATGCGCGCTCTTCAT	942 bp
At5g02380	SALK_144899	GAGGAACGATTATTTAGGTG	AGAGAAAGACCAACGTCGCC	713 bp
At5g67300	SALK_039074	TTGCGTGGAAGAAGCGTTGC	ATTCGTTGACTCGTGGCTAC	839 bp
At5g67310	GK_820-E01	AAGAGCTCCGAGCAAACAAC	CCTACAGGATTTGGCGGTAA	433 bp
At5g16930	SAIL_229_H04	ACCCTACTAAAACCTTTGGC	TGTTAGAATGGTACGCAACC	1746 bp
At5g16940	SALK_067777	TCTCATTCTCAGTTCCCCAC	GTCCTTTGAGTCATCAAGAAG	676 bp

SALK insertion lines the primer: 5′-AACCAGCGTGGACCGCTTCTG-3′

SAIL lines primer: 5′-TAGCATCTGAATTTCATAACCAATCTCGATACAC-3′

Gabi-Kat lines primer: 5′-CCCATTTGGACGTGAATGTAGACAC-3′.

### Poly(A) analysis

For 3′ RACE analysis cDNA was prepared from 5 µg of total RNA using 3′RACE primer 1575. The 3′end of short At5g67300 sense transcripts was amplified using primers 2116 and 1576. The 3′ end of the long At5g67300 sense transcripts was amplified by semi-nested PCR using primers 2015 and 1576 for primary amplification, and primers 2214 and 1576 for secondary amplification.

Similarly, the 3′ end of At5g67310 antisense transcripts was amplified by semi-nested PCR using primers 2215 and 1576 in the first PCR reaction, and primers 2225 and 1576 in the second PCR reaction.

The 3′ end of At5g02370 sense short transcripts was amplified in two PCR reaction using primers 2197 and1576 in the primary PCR and primers 2118 and1576 in the secondary PCR. To amplify long At5g02370 transcripts, the first PCR was performed with primers 2018 and 1576, and the second PCR with primers 2201 and1576. The 3′ end of At5g02380 antisense transcripts was amplified with primers 2019 and 1576.

The 3′end of At5g16930 sense transcript was amplified using primers 1969 and1576, and the At5g16940 antisense transcript was amplified by semi-nested PCR using primers 1164 and1576 for the primary amplification, and primers 1160–1576 for the secondary PCR. PCR fragments were cloned into pGEM-T Easy vector system (Promega) according to the manufacture's instruction and were sequenced.

### Transient assay

Protoplasts were transformed by PEG-mediated transfer [40]. Samples were collected 6 h after protoplasts incubation. RNA was isolated using RNeasy plant Mini kit (QIAGEN).

### Isolation of polysomal RNA from plant tissue

Polysomes were isolated from flowers [41] RNA from polysomes was isolated using the RNeasy plant Mini kit (QIAGEN).
